# First Oral Vaccination of Eurasian Wild Boar Against African Swine Fever Virus Genotype II

**DOI:** 10.3389/fvets.2019.00137

**Published:** 2019-04-26

**Authors:** Jose A. Barasona, Carmina Gallardo, Estefanía Cadenas-Fernández, Cristina Jurado, Belén Rivera, Antonio Rodríguez-Bertos, Marisa Arias, Jose M. Sánchez-Vizcaíno

**Affiliations:** ^1^Animal Health Department, Faculty of Veterinary, VISAVET Health Surveillance Centre, Complutense University of Madrid, Madrid, Spain; ^2^European Union Reference Laboratory for ASF, Centro de Investigación en Sanidad Animal (INIA-CISA), Madrid, Spain; ^3^Department of Animal Medicine and Surgery, Faculty of Veterinary, Complutense University of Madrid, Madrid, Spain

**Keywords:** attenuated ASF virus, control disease, Eurasian wild boar, oral immunization, protective vaccine

## Abstract

African swine fever (ASF), the most significant threat to the pig industry worldwide, has spread to more than 55 countries on three continents, and it affects more than 77% of the world swine population. In the European Union, wild boar (*Sus scrofa*) is the most severely affected host. The main reasons for the unprecedented and constant spread of ASF in Europe are the trade activities, the continuous movement of infected-wild boar populations among regions and the lack of vaccine to prevent ASF infection. In this study, we demonstrate that oral immunization of wild boar with a non-hemadsorbing, attenuated ASF virus of genotype II isolated in Latvia in 2017 (Lv17/WB/Rie1) conferred 92% protection against challenge with a virulent ASF virus isolate (Arm07). This is, to our knowledge, the first report of a promising vaccine against ASF virus in wild boar by oral administration. Further studies should assess the safety of repeated administration and overdose, characterize long-term shedding and verify the genetic stability of the vaccine virus to confirm if Lv17/WB/Rie1 could be used for free-ranging wild boar in ASF control programs.

## Introduction

African swine fever (ASF) is one of the most complex and devastating viral diseases affecting suids. Virulent ASF virus (ASFV) strains cause peracute or acute hemorrhagic fever in infected animals with up to 100% mortality (1). Due to the devastating socioeconomic and animal health consequences, ASF is listed as a notifiable disease by the World Organization for Animal Health. After its introduction from Eastern Africa into Georgia (2), ASFV genotype II has been circulating in Eastern Europe since 2007, in the European Union since 2014 and in Asia since 2018 (3). Despite control measures, notifications continue to arrive from wild boar (*Sus scrofa*) and domestic pig farms (3). Neither a vaccine nor specific treatment is available against ASFV. Control measures include depopulation of affected domestic and wild populations, as well as movement restrictions on trade of live pigs and derived products at regional, national and international levels (1, 4, 5). Thus, ASF represents the most significant threat to the current pig industry worldwide (6, 7).

Currently, ASF affects more than 55 countries on 3 continents, including China, which contains nearly half of the world's pig population. The epidemiology of ASF varies significantly depending on the characteristics of the circulating virus strain, the presence of wild hosts and reservoirs, farm biosecurity, environmental conditions, and human behavior (4, 8). Nine members of the European Union have reported ASF in the last 5 years: Lithuania, Poland, Latvia, Estonia, Czech Republic, Romania, Hungary, Bulgaria, and Belgium (3). In all these countries except Romania, wild boar is the main host affected by the disease, accounting for more than 90% of outbreaks in the Union (3). An epidemiological analysis in Estonia concluded that the presence of ASF in wild boar is the main risk factor for domestic pig outbreaks (9).

Transboundary movements of ASF occur as a result of illegal movements of infected pigs, the introduction of pork or pork products contaminated with ASFV, and swill feeding practices (1, 10). While these remain the major risk factors for ASF spread across long distances (2, 11), local maintenance and spreading of infection can occur through movements of free-ranging infected wild boar *via* natural connected landscapes, which keeps the virus endemic on wild boar populations in the European Union (9, 12).

ASF occurs in several clinical forms that can range from peracute to subclinical. Clinical manifestations depend on isolate virulence, host, dose, and route of infection, among other factors. The incubation period ranges from 3 to 19 days (13). Clinical signs and lesions involve congestive, hemostatic, and hemodynamic changes such as hemorrhage, edema, ascites, and shock, as well as functional disorders of digestive and respiratory systems (8). The range of mortality varies from 10 to 100%, depending on the virulence of the isolate (1). ASFV genotype II strains responsible for Eurasian outbreaks are highly virulent and induce an acute clinical form associated with nearly 100% mortality in domestic pigs and wild boar (14–17). Recent reports also suggest that moderately virulent ASFV strains are circulating in Europe (18, 19).

Overall, vaccine development has been hindered by ASFV genetic complexity, gaps in knowledge concerning ASFV infection and immunity, lack of development of neutralizing antibodies, and technical difficulties such as the lack of stable cell lines. In fact, vaccine development has been identified as a major gap in ASF control and eradication (13).

The recent re-emergence of ASF in Europe has increased interest in the development of an effective vaccine against ASF. Attempts to vaccinate animals using inactivated virus or subunit vaccines have failed to induce protective immunity (20–22). Live vaccines attenuated by serial passage in cell culture or through genetic deletion can induce partial or full protection (20, 23). Engineering of attenuated vaccine candidates is facilitated by substantial progress in identifying ASFV genes involved in virulence and immune evasion. In fact, several ASFV genome sequences are now available (24). Naturally attenuated live vaccine candidates can also be isolated from the field (25). A weakly virulent, non-hemadsorbing ASFV strain was isolated in 2017 from a hunted wild boar in Latvia (Lv17/WB/Rie1) (25). Experimental infection of domestic pigs with this strain provided complete protection against a virulent hemadsorbing ASFV genotype II, suggesting the potential use of Lv17/WB/Rie1 as a live attenuated vaccine (25).

Despite the great interest in an ASFV vaccine for wild boar, we are aware of vaccination trials only in domestic pigs (see bibliographic review in Supplementary Table 1). The importance of vaccinating wild boar was demonstrated during the 2000's when classical swine fever affected different European countries (26). The aim of this experimental study was to assess how well oral immunization of wild boar with the Lv17/WB/Rie1 strain would protect them against challenge with a virulent ASFV genotype II isolate (Arm07).

## Materials and Methods

### Animals

Eighteen female wild boar piglets 3–4 months old and weighing 10–15 kg were obtained from a commercial wild boar farm in Extremadura, Spain. Piglets had not been vaccinated against any infectious disease. Wild boar from this site tested negative for the following main porcine pathogens in the region: Aujeszky virus, *Mycobacterium bovis, Mycoplasma pneumoniae* and porcine circovirus type 2. Piglets were kept at the BSL-3 biocontainment facilities of the VISAVET Centre at the University Complutense of Madrid. Animals were allowed to acclimate for 2 weeks before experiments began. During the trial, wild boar had *ad libitum* access to food and water. All experiments were carried out following European, national and regional regulations and were approved by the Ethic Committee of Comunidad de Madrid (reference PROEX 124/18).

### ASFV Isolates

The attenuated, non-hemadsorbing p72 genotype II ASFV Lv17/WB/Rie1 was used for vaccination. This strain had previously been tested in domestic pigs (25). The distinctive non-hemadsorbing phenotype is related to a unique adenosine clearance that generates a truncated protein from the CD2-like coding sequence in the *EP402R* gene (Spanish patent PCT/2018/000069). This deletion corresponds to the gene position 395 in the hemadsorbing ASFV Georgia 2007/1 reference genome (GenBank FR682468). Virus was grown for 7 days in porcine blood monocytes (26), then the culture medium containing extracellular virus was collected, centrifuged at low speed to remove cellular debris and then at high speed to sediment the virus. The sediment was suspended in phosphate-buffered saline (PBS), titrated in porcine blood monocytes, and used in protection experiments. Viral titer was defined as the amount of virus causing cytopathic effects in 50% of infected cultures (TCID50/ml), as estimated by immunoperoxidase staining.

For challenge experiments, the virulent, hemadsorbing ASFV genotype II strain Arm07 was used (23). Virus was propagated in porcine blood monocytes as described (23). Viral titer was defined as the amount of virus causing hemadsorption in 50% of infected cultures (HAD50/ml/TCID50/ml).

### Wild Boar Immunization and Challenge

Twelve wild boar piglets were hosted at the BSL-3 facilities to conduct the vaccination trial. Initially, nine wild boar were orally vaccinated with 10^4^ TCID50 of Lv17/WB/Rie1 ASFV. Subsequently, the remaining three wild boar were exposed to the orally vaccinated piglets through contact (hereafter called VContact) from 0, 7, and 15 days after vaccination to test the vaccine transmission at different times.

The vaccination period lasted 30 days to allow development of an immune response. Then a shedder-pig challenge-exposure infection model was used to assess protective immunity: vaccinated animals were exposed to four naïve wild boar (shedder animals). These naïve animals were intramuscularly inoculated with 10 HAD50 of ASFV Arm07 on the same day as intramuscularly challenged controls. Also at 30 days after vaccination, two naïve wild boar were used as late in-contact animals: the naïve animals were exposed to all other animals, and the transmission of the vaccine or the challenge virus was measured.

All 12 vaccinated animals, four wild boar intramuscularly challenged with Arm07, and two naïve late in-contact wild boar were maintained for 24 days after challenge, corresponding to 54 days after vaccination.

During this time, animal motion was monitored 24 h a day by video camera. Clinical signs were recorded on a daily basis as described (5, 23); these signs included anorexia, recumbence, skin hemorrhage or cyanosis, joint swelling, respiratory distress, ocular discharge and digestive findings. Paired EDTA-blood and serum samples were collected twice a week. Rectal temperature was measured twice a week prior sampling animals as well as in animals showing any clinical signs after vaccination. Presence of ASFV genome in blood was determined using real-time PCR (27). Serum samples were assayed for antibodies using a commercial ELISA test (Ingenasa-Ingezim PPA Compac K3; Ingenasa, Madrid, Spain) and using an indirect immunoperoxidase test (IPT) (13).

### Necropsy and Sample Collection

At the end of the observation period (54 days after vaccination), survivor animals were anesthetized by intramuscular injection of an anesthetic combination of tiletamine-zolazepam (Zoletil® 100 mg/ml, Virbac, France, target dose 3 mg/kg) and medetomidine (Medetor®, Virbac, France, target dose 0.05 mg/kg) (28), then euthanized by intravenous injection of T61. A thorough post-mortem examination was done to detect the presence of macroscopic lesions compatible with ASF. Sixteen different tissues (listed in Table 1), including all major lymph nodes and target visceral organs, were obtained from each necropsied animal and tested by real-time PCR to detect ASFV. Virus isolation was performed from a subset of tissues, using established procedures (29). Samples were blind-passaged three times, and plates were examined for hemadsorption during 6 days. If ASF-compatible lesions were found, they were classified based on their distribution and intensity.

**Table 1:** List of post-mortem tissues tested for African swine fever virus DNA using real-time PCR.

**Monitored tissues**	
Bone marrow	Mediastinal lymph node
Brain	Mesenteric lymph node
Gastrohepatic lymph node	Prescapular lymph node
Heart	Renal lymph node
Inguinal lymph node	Retropharyngeal lymph node
Kidney	Spleen
Liver	Submandibular lymph node
Lung	Urinary bladder

### Statistical Analysis

Kaplan-Meier survival curves and the Mantel-Cox log rank test were used, respectively, to compute probability of death and to test for significant survival differences among groups. The Mann-Whitney *U* test and Spearman's rank correlation were used to compare Ct values from real-time PCR among treatment groups. Data were analyzed using SPSS 20 (IBM, Somar, NY, USA) at a significance level of 0.05.

## Results

### Outcomes During Vaccination Period

**Figure 1:**
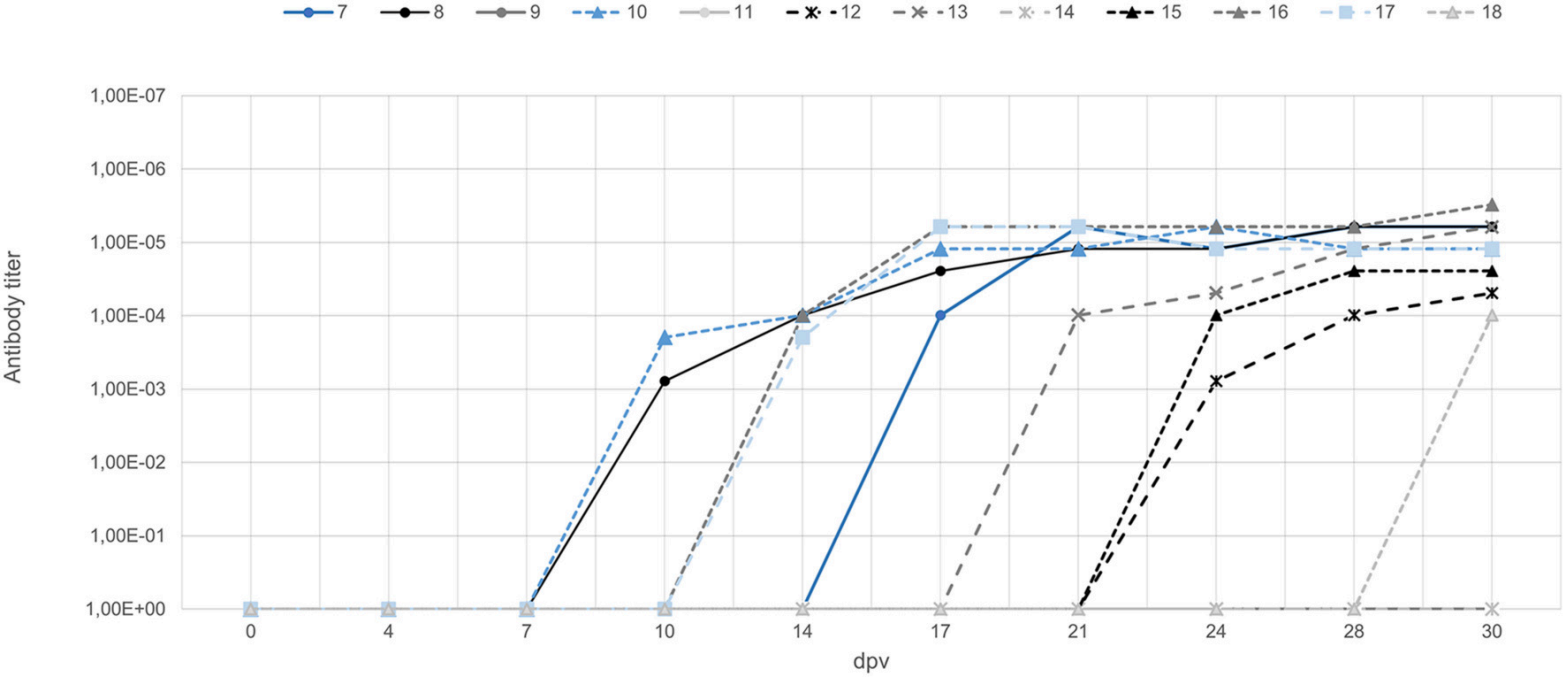
Titers of antibody against ASFV in wild boar orally vaccinated with Lv17/WB/Rie1 (gray) and wild boar exposed through contact with vaccinated animals (blue). The latter animals were exposed through contact starting from 0 days (animal ID7), 7 days (ID10), and 15 days (ID17) after vaccination. Titers were determined using the indirect immunoperoxidase test.

During the 30-day vaccination period, six of nine orally vaccinated animals were positive for anti-ASFV antibodies based on ELISA and IPT tests starting from 15 ± 3 days after vaccination (Figure 1). All three VContact wild boar showed positive antibody response starting at 14 ± 2 days after contact, and titers remained high throughout the experiment (Figure 1). These results indicate that orally administered Lv17/WB/Rie1 strain can induce an antibody response in wild boar.

**Figure 2:**
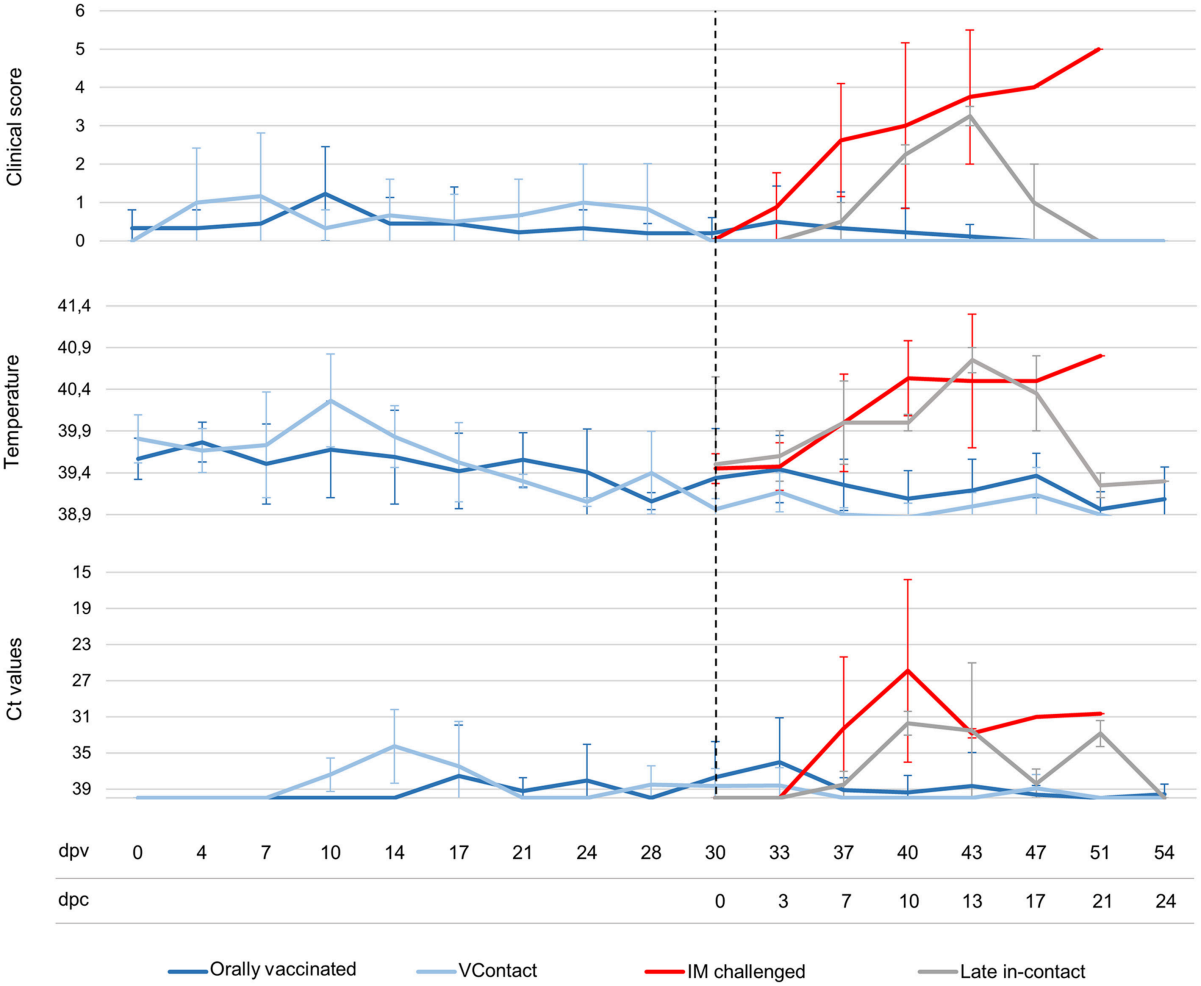
Average of clinical scores, body temperatures and Ct values from real-time PCR of wild boar orally vaccinated with Lv17/WB/Rie1 (*n* = 9; dark blue), exposed through contact (VContact, *n* = 3; light blue), intramuscularly challenged controls inoculated with virulent ASFV Arm07 (*n* = 4; red) and late in-contact wild boar (*n* = 2; gray). Averages are shown at different days post-vaccination (dpv), including days post-challenge (dpc). Error bars indicate SD.

No ASF-compatible clinical signs were detected in animals immunized with Lv17/WB/Rie1. The only clinical reaction detected was a slight increase in body temperature to 40.1–40.8°C in seven of nine vaccinated animals and in one of three VContact animals, which lasted an average of 3.5 days between 4 and 24 days after vaccination (Figure 2). Viremia peaked on different days in the animals. In six of nine orally vaccinated animals and two of three VContact wild boar, real-time PCR sporadically showed weakly positive results (Ct = 33.02 ± 4.07) during the 30-day vaccination period. Viremia peaks showed a weak correlation with the slight increase in body temperature (Figure 2).

### Outcomes After Challenge

The immune response in vaccinated and VContact animals protected against Arm07. After challenge-exposure, 11 of the 12 vaccinated and VContact animals survived (92%). Moreover, none of them developed any ASF-compatible clinical signs or gross lesions after challenge. Two orally vaccinated animals that had shown neither anti-ASFV antibody response or an increase in body temperature during the 30-day vaccination period developed intermittent viremia peaks after challenge. They showed positive antibody response at 3 and 7 days after challenge, corresponding to 33 and 37 days after vaccination.

In contrast, all controls that received intramuscular challenge developed severe clinical signs compatible with ASF (Figure 2). These animals died or were euthanized between 7 and 20 days post-infection (Mantel-Cox, χ^2^ = 18.88, 1 d.f.; *p* < 0.001). The two late in-contact animals showed clinical signs similar to those of intramuscularly challenged controls (Figure 2), except that the late in-contact animals developed antibody responses at 7 and 9 days after challenge, and subsequently recovered their pre-challenge state and survived. The one orally vaccinated animal that did not survive the challenge developed a clinical form of ASF similar to that observed in intramuscularly challenged controls. This wild boar never showed an antibody response.

All intramuscularly challenged control animals showed viremia starting 6–12 days after challenge until death (Ct = 23.65 ± 4.68). The two late in-contact animals showed viremia from 6 or 11 days after challenge until 21 days (Ct = 32.74 ± 1.11). Four of eight orally vaccinated surviving animals and one of three VContact wild boar sporadically showed weakly viremia peaks after challenge (Ct = 34.56 ± 1.60). The animal orally vaccinated and unprotected against challenge developed viremia of similar Ct values as intramuscularly challenged controls (Ct = 26.31 ± 1.73). In general, viremia Ct values from real-time PCR were significantly higher in surviving animals than in this unprotected animal or in intramuscularly challenged controls (Mann-Whitney *U* test, *Z* = −2.84, *p* < 0.01) (Figure 2).

**Figure 3:**
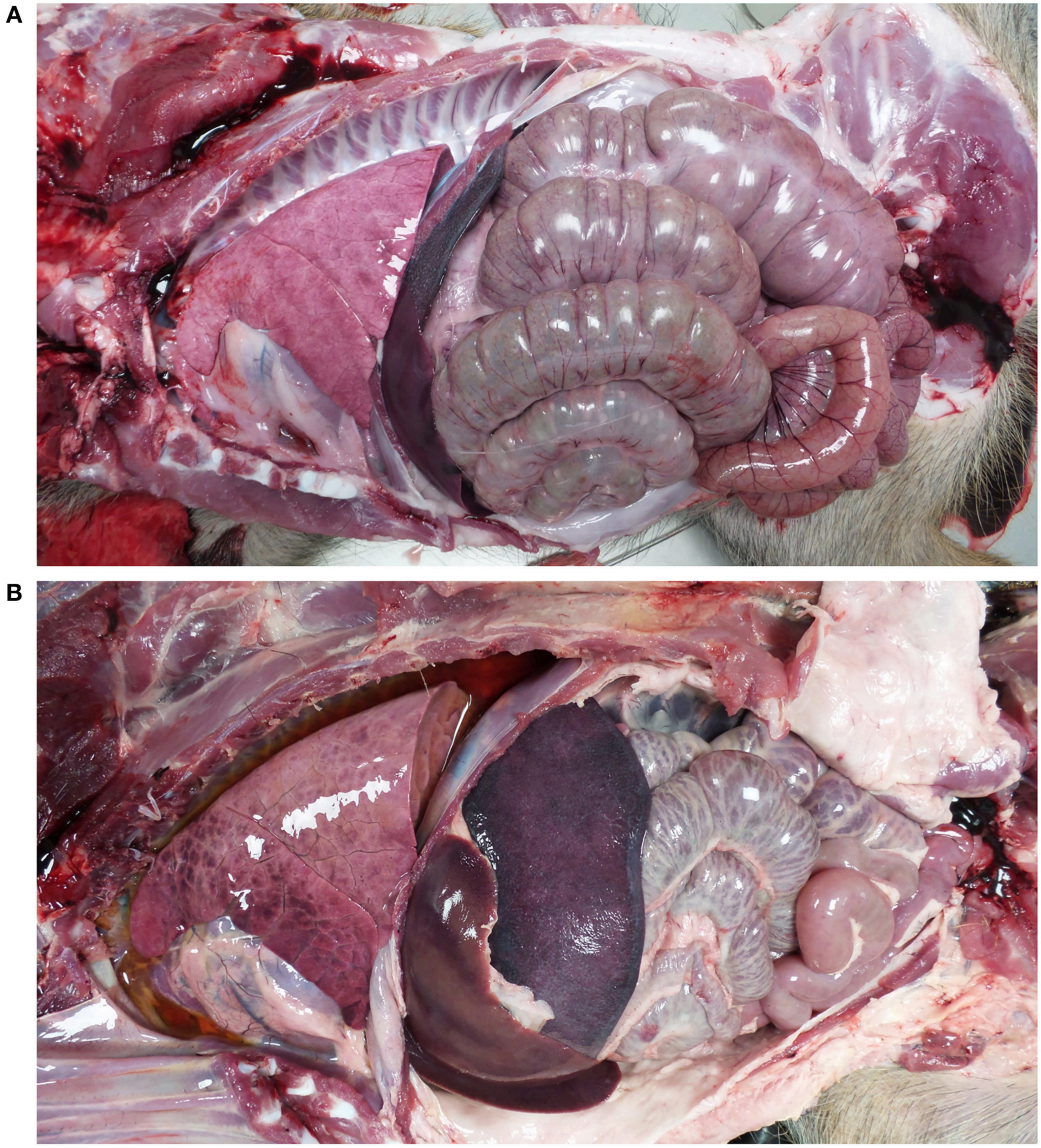
Views of thoracic and abdominal cavities from **(A)** wild boar orally vaccinated with Lv17/WB/Rie1 and **(B)** intramuscularly challenged control with virulent ASFV Arm07. Hydrothorax, hepatomegaly, and splenomegaly are evident in **(B)**.

**Figure 4:**
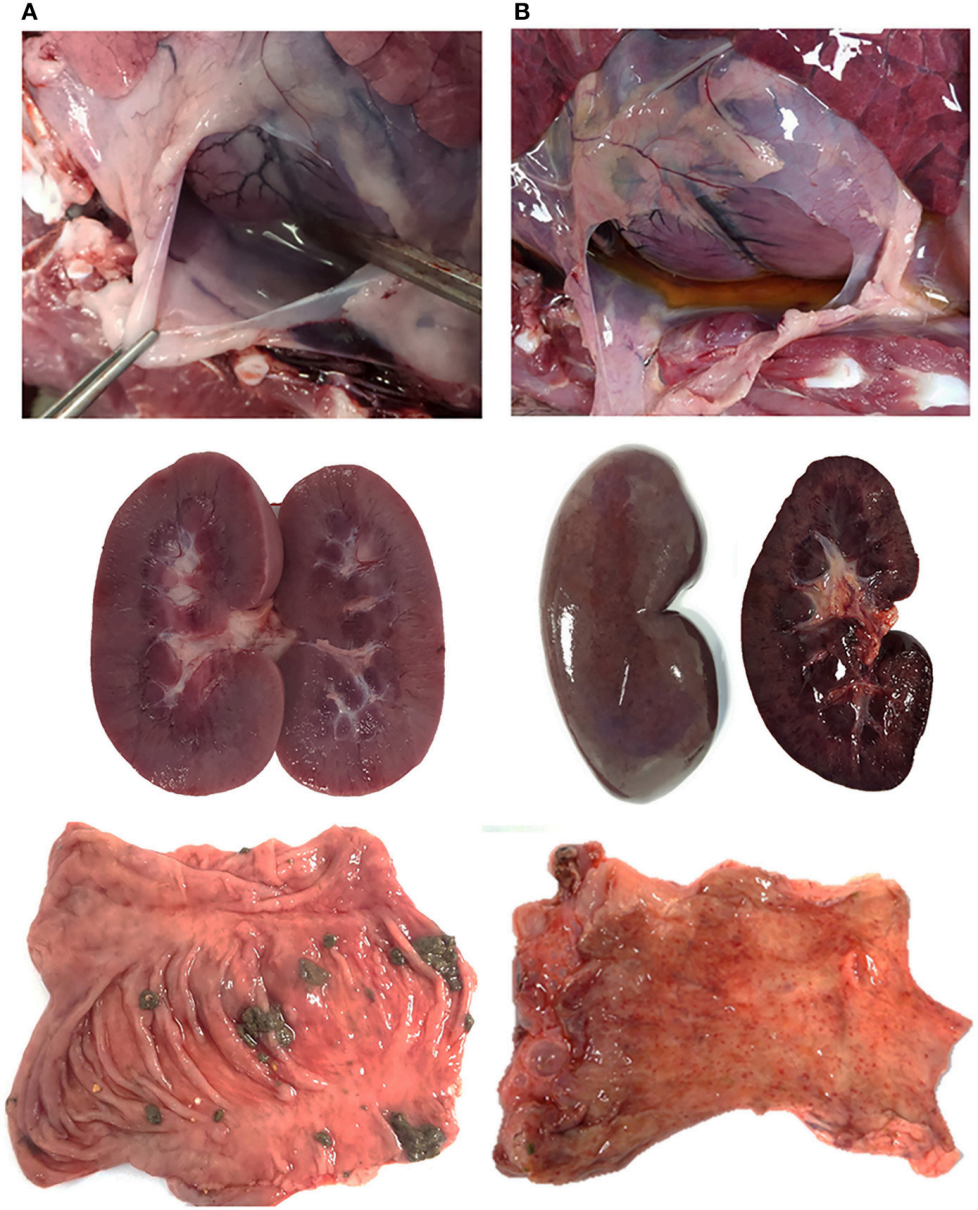
Survey view of pericardial sac (I), kidney (II), and mucosal surface of the intestine (III) from **(A)** wild boar orally vaccinated with Lv17/WB/Rie1 and **(B)** intramuscularly challenged controls inoculated with virulent ASFV Arm07. **(B)** Shows hydropericardium (IB), congestive kidney with acute multifocal ranging from petechial to ecchymotic hemorrhages on the cortex surface (IIB), and numerous acute petechiae on the mucosal surface of the colon (IIIB).

Post-mortem analyses revealed ASF-compatible pathological findings only in the vaccinated unprotected animal and intramuscularly challenged controls. The main necropsy findings were moderate to severe accumulation of fluid from yellowish to reddish in the abdominal cavity (ascites), in the thorax (hydrothorax) and in the pericardial sac (hydropericardium). General congestion and focal hemorrhages were observed on the lung surface, spleen (splenomegaly), lymph nodes (hemorrhagic lymphadenitis), kidneys, vesical mucosa (diffuse hemorrhagic cystitis), and gastric mucosa (Figures 3, 4).

**Figure 5:**
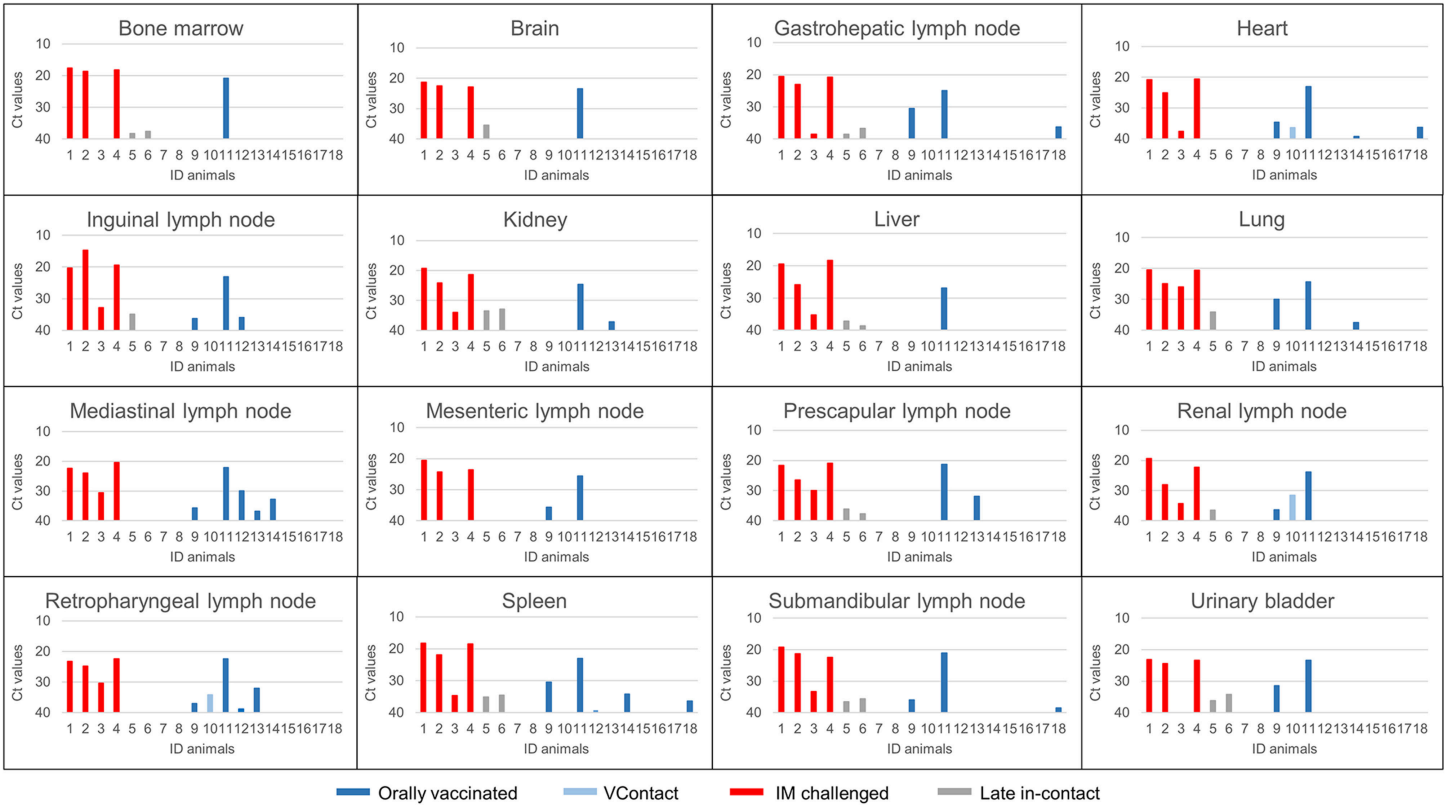
ASFV DNA levels (Ct values from real-time PCR) in post-mortem tissues from animals orally vaccinated with Lv17/WB/Rie1 (dark blue), exposed through contact (VContact, light blue), intramuscularly challenged controls inoculated with virulent ASFV Arm07 (red) and late in-contact wild boar (gray).The unique orally vaccinated animal that did not survive the challenge (ID11) gave strongly positive results in several tissues (Ct = 23.32 ± 1.60), similar to the intramuscularly challenged controls.

**Figure 6:**
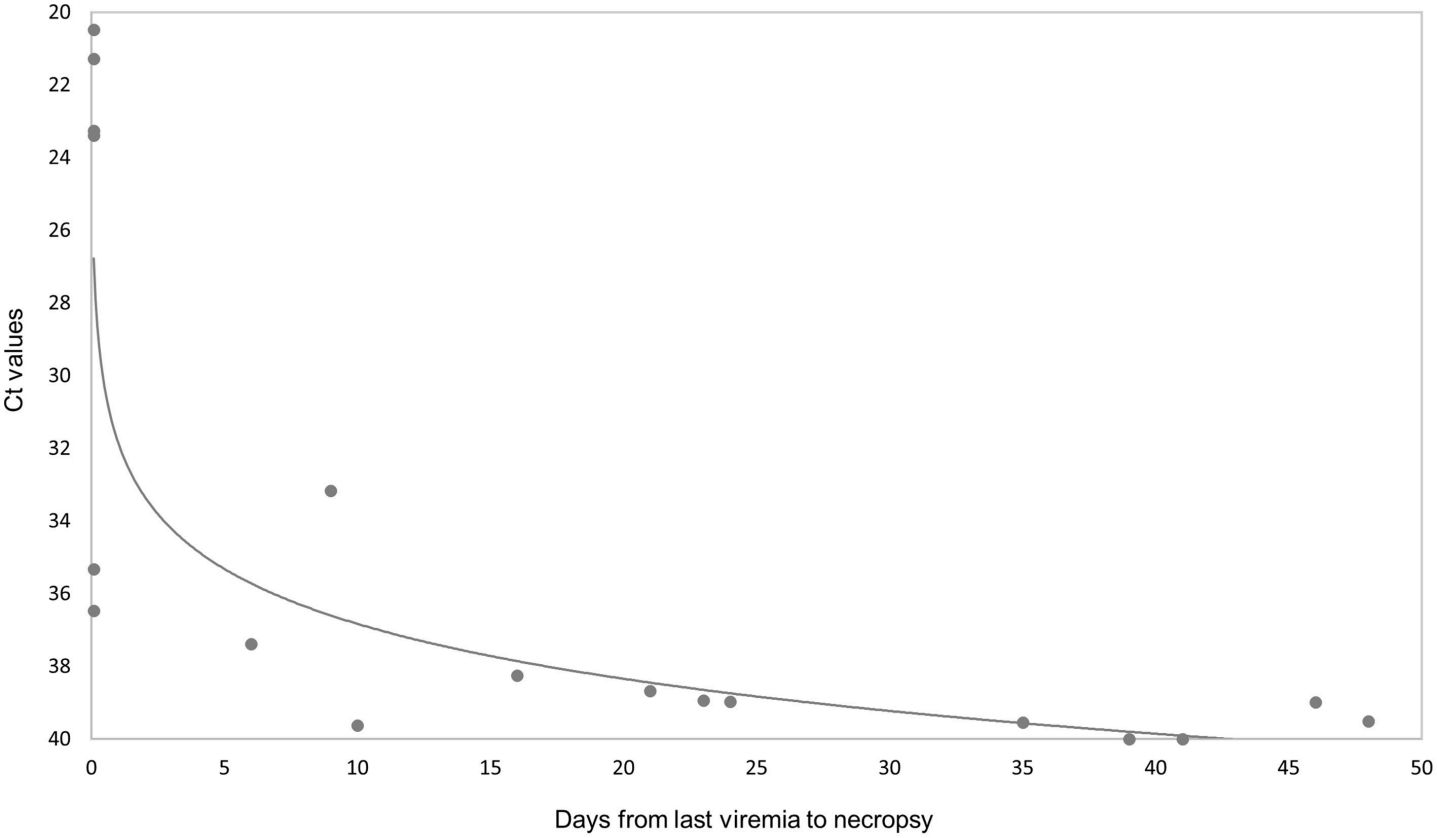
Comparison of ASFV DNA levels (Ct values from real-time PCR results) in 16 post-mortem tissues with the number of days from last viremia detected until necropsy. The Ct values are inversely related to the level of virus genome.

ASFV genomic DNA was not detected in any of the 16 tissues analyzed in three of eight orally vaccinated surviving animals and two of three VContact animals. The remaining animals that survived showed weakly positive PCR results (Ct = 38.416 ± 1.16) in an average of 5 tissues. ASFV could be isolated from only two of 22 tissues analyzed from these animals: retropharyngeal lymph node in one vaccinated animal, and renal lymph node in one VContact animal. These virus isolates were non-hemadsorbing. The two late in-contact wild boar showed weakly positive PCR results in 9 or 12 tissues (Ct = 37.40 ± 0.43), where hemadsorbing ASFV was isolated only from one inguinal lymph node. In contrast, viral DNA was detected in almost all 16 tissues analyzed from four intramuscularly challenged animals, and levels were significantly higher (Ct = 21.59 ± 1.26) than in animals that survived (Mann-Whitney *U* test, *Z* = −2.65, *p* < 0.01). In this case, hemadsorbing ASFV was isolated from several tissues. In a similar way, the one orally vaccinated animal that remained unprotected against challenge showed strongly positive PCR results (Ct = 23.32 ± 1.60) in all 16 tissues tested. These results are summarized by tissue and animal in Figure 5. Viral genome load correlated inversely with the interval between last viremia detected and post-mortem analysis (Spearman's rank correlation, *r* = −0.853, *p* < 0.001; Figure 6).

## Discussion

Under experimental conditions, a new field naturally attenuated ASFV isolate (Lv17/WB/Rie1 strain) (25) was tested as vaccine candidate for wild boar. As the target of this vaccine would be wild boar in field, we considered of great interest to use such prototype vaccine by oral administration, demonstrated in past successful experiences (i.e., oral immunization against classical swine fever of wild boar in Germany)(30). Our results showed that the Lv17/WB/Rie1 strain protected 92% of orally vaccinated and VContact animals against challenge with the virulent Arm07 isolate. This protection translated not only to animal survival but also to the absence of ASF-compatible clinical signs, pathological findings, and virus detection in target tissues. The vaccine candidate would be the first oral vaccine against ASFV genotype II tested in wild boar. The potential use of this vaccine in field would aim at reducing the number of susceptible animals, increasing herd immunity in wild boar populations, and so, decreasing ASF incidence.

This complete *in vivo* study provides details on clinical and pathological findings, antibody responses, viremia periods and DNA virus detection in 16 target tissues and organs. When comparing these results to previous experimental studies of candidate vaccines tested in domestic pigs, it is highlighted the protective efficacy against a non-parental virulent challenge ASFV genotype II (see Supplementary Table 1). Although our results were obtained with unbalanced size of animal groups, the high protective effect in wild boar observed in this study are consistent with previous results obtained with Lv17/WB/Rie1 in two domestic pigs inoculated intramuscularly with this isolate and four in-contact animals (25). Therefore, this natural isolate appears to be more effective than other strains that have been attenuated through successive passages in cells (31) or by genetic manipulation which have challenged against the non-parental viruses (32–34). In this sense, further studies on cross-protective effects of Lv17/WB/Rie1 are needed against different ASFV genotypes, since the virulent ASFV Arm07 used as challenge in this study also belongs to the genotype II.

There are many studies on obtaining live attenuated ASF vaccines by genetic engineering, it can be considered as a potential tool to improve our candidate vaccine, allowing us to delete virulent genes to maximize safety. Nevertheless, we have to keep in mind that some genes have an essential protective role and should be maintained. As the case of the attenuated ASFV NH/P68 strain, it confers total protection against the Arm07 isolate, but after genetic manipulation, the deletion mutants assayed to date did not demonstrate their ability to fully protect against the challenge with heterologous virulent virus strain, reducing the efficacy of such candidates (23).

Our observation that three wild boar were immunized through contact indicates that orally vaccinated animals can shed vaccine virus. This shedding, which has also been described for the attenuated ASFV NH/P68 vaccine candidate (23), may help amplify vaccination coverage, reducing the need for expensive production and large-scale administration of vaccine in the field.

On the other hand, shedding of the vaccine virus can mean that wild boar that recover from ASF act as virus carriers and ensure persistent infection (18, 19, 35, 36). However, two long-term studies showed that after clinical recovery from ASF, animals infected with moderately virulent ASFV were able to eliminate the virus from serum and tissues and did not transmit the virus to commingled sentinel pigs (37, 38). In fact, the surviving animals showed high antibody titers for more than 3 months after initial infection (37). These results agree with those in our study, where orally vaccinated and VContact wild boar maintained high antibody titers (Figure 1). While viremia (Figure 2) and presence of viral DNA in tissues (Figure 6) decreased during the experiment, even after exposition to virulent challenge. Our isolation of non-hemadsorbing Lv17/WB/Rie1 from only two tissues from all orally vaccinated and VContact animals at the end of the experiment suggests low risk of infectivity after viremia periods (25), although we consider this result preliminary since we did not attempt to isolate virus from all available tissues. In addition, long-term studies are urgently needed to evaluate the ability of Lv17/WB/Rie1 to persist and be transmitted among sentinel wild boar. This is especially important given contradictory studies about the ability of animals to act as ASFV carriers, which may depend on virus strain (18, 19, 35–38). Further studies are also needed to assess the safety of Lv17/WB/Rie1 as a vaccine, such as to establish what happens when animals receive an overdose and to examine virus shedding routes over time (20, 39, 40).

Our inability to detect ASFV DNA by PCR in three of eight orally vaccinated surviving animals and two of three VContact animals during post-mortem tissue analysis suggests that these animals had completely eliminated the virus. The remaining animals that survived showed weakly positive PCR results mainly in retropharyngeal and submandibular lymph nodes, indicating that the animals had not eliminated the vaccine virus or that they had likely eliminated it but were reinfected by virus that persisted in the animal pens from previous viremia periods. This virus was most likely Lv17/WB/Rie1 because all isolates from these surviving animals were non-hemadsorbing. In contrast, intramuscularly challenged controls and one vaccinated unprotected wild boar showed strongly positive PCR results in all tissues analyzed (see Figure 5), and the viral DNA was most likely that of the challenge strain Arm07, since viral isolates were hemadsorbing. These findings suggest that Lv17/WB/Rie1 virus can be eliminated and is not efficiently transmitted in the long term, at least at the dose and administration route in this study. Consistent with this idea, viral DNA levels correlated inversely with the interval between last viremia and necropsy (Figure 6).

Our analysis suggests that vaccination helped the two late in-contact wild boar recover from ASFV infection. Initially after challenge, their viremia, temperature and clinical signs were similar to those of intramuscularly challenged animals (Figure 2), and hemadsorbing ASFV Arm07 was even isolated from the inguinal lymph node of one late in-contact animal. Subsequently, though, the two late in-contact animals showed high antibody response at 7–9 days after challenge and decreased clinical signs and viremia (Figure 2). Our observation that both late in-contact animals showed presence of ASFV DNA in tissues similarly to orally vaccinated and VContact surviving animals (Figure 5) likely reflects the fact that the late in-contact animals were exposed to both isolates at the same time. In fact, the clinical recovery and elimination of virus in late in-contact animals suggest that Lv17/WB/Rie1 can be a highly effective vaccine, given that it conferred protection even in the presence of the virulent Arm07 isolate. These results should be verified and extended in further studies of animal exposure to both challenge and vaccine virus.

To the best of our knowledge, this is the first report of experimental vaccination of wild boar against ASFV genotype II, and the first report of oral immunization against any ASFV strain in wild boar. In the current context of this transboundary disease, an oral vaccine against ASFV in wild boar is urgently needed as an additional tool to re-inforce and re-design mitigation plans owing that none of the control measures applied in affected wild boar populations has been effective (7, 13, 41). If the safety of Lv17/WB/Rie1 as a vaccine can be established, then it may help mitigate the uncontrolled spread of ASFV across Europe, similar to the success so far in halting the spread of classical swine fever. Future studies should examine the vaccine's safety following repeated administration or overdose, its genetic stability during passages, its stability in the field, and its differentiability from infecting virus based on DIVA serological testing.

## Author Contributions

JB, CG, EC-F, MA, and JS-V: participated in experimental design. CG, BR, and MA: prepared the vaccines. JB, CG, EC-F, CJ, BR, AR-B, and JS-V: conducted field and laboratory work. JB, EC-F, CJ, BR, and JS-V: performed data analysis. JB, EC-F, and CJ: drafted the manuscript. CG, BR, AR-B, MA, and JS-V: revised the manuscript.

### Conflict of Interest Statement

The authors declare that the research was conducted in the absence of any commercial or financial relationships that could be construed as a potential conflict of interest.
