# Supplementary material for: First Oral Vaccination of Eurasian Wild Boar Against African Swine Fever Virus Genotype II
Source: Front Vet Sci. 2019 Apr 26;6:137. doi: 10.3389/fvets.2019.00137 (PMC6498142; doi:10.3389/fvets.2019.00137)
Supplement: Supplementary file 1 [file Table_1.DOCX]

1. **Supplementary table 1.** Table of bibliographic review of the most relevant studies about immunisation tests against African swine fever virus.

| **Bibliographic references** | **Animal** | **Type of vaccine** | **Vaccinal strain or vaccinal construction** | **Vaccinal route** | **Vaccinal dose** | **Challenge strain** | **Challenge dose** | **DPV Challenge** | **Immune response** | **Protection against challenge** | **Clinical signs before challenge (attenuation of the vaccine)** |
| --- | --- | --- | --- | --- | --- | --- | --- | --- | --- | --- | --- |
| King et. al (2011) | Domestic pigs | Live attenuated isolated | OURT88/3 and boost with OURT88/1 | IM | 10^4 TCDI50 and 10^4 HAD50 boost 21 dpv | Benin 97/1 | 10^4 HAD50 | 42 | 100% Ab ASFV ELISA | 85,70% | No clinical signs (n=13), transient pyrexia (n=5), severe ASF symptoms (n=4) |
|  |  |  |  |  |  | Uganda 1965 |  |  |  | 100% |  |
| Argilaguet et. al (2012) | Domestic pigs | Recombinant vaccine | pCMV-sHAPQ (encoding: p54, p30, extracelular domain viral hemagglutinin | IM | 4 doses 15-day intervals | E75 | 10^4 HAU50 |  | Antibodies and specific T cell responses against p30 and p54 | 0% | - |
| Lacasta et. al (2014) | Domestic pigs | Recombinant vaccine | ASFVUblib or pCMV-Ub | IM | 10^4 HAU | E75 | 10^4 HAU50 | 28 | 0% Ab ASFV ELISA prior challenge. CD8  T-cell responses prior to ASFV challenge | 60% |  |
| Krug et. al (2014) | Domestic pigs | Attenuated vaccine by successive passeges in cells | ASFV-G/VP30, ASFV-G/VP60, ASFV-G/VP80, ASFV-G/VP110 | IM | 10^4 HAD50 | ASFV-G (Georgia 2007/1) | 10^4 HAD50 | 21 and 28 | - | 0% | Severe ASF symptoms (except animals inoculated with ASFV-G/VP110-no clinical signs) |
|  |  |  |  |  | 10^2 HAD50 |  |  |  | - | 0% | Transient disease (ASFV-G/VP30). No clinical signs (ASFV-G/VP60, ASFV-G/VP80, ASFV-G/VP110) |
| Souto et. al (2014) | Domestic pigs | Live attenuated isolated | OURT 3/88 | IM | 10^4 HAD50 | MOZ 1/98 | 10^4 HAD50 | 49 |  | 0% (difference in survival times) | No clinical signs |
|  |  |  |  |  | 10^4 HAD50 and boost 21 dpv |  |  |  |  |  |  |
| Mulumba-Mfumu et. al (2015) | Indigenous breeds of pigs | Live attenuated isolated | OURT88/3 | IM | 10^4 TCDI50 | OURT88/1 | 10^4 HAD50 | 21 |  | 50% | Slightly elevated temperature  between 39.1 and 40°C over the first 9 or 10 days |
|  |  |  |  |  |  | DRC  strain 085/10 |  | 42 |  | 100% of the animals which had survived OURT88/1 challenge |  |
| O’Donnell et. al (2015a) | Domestic pigs | Attenuated vaccine by genetic manipulation | ASFV-GΔ9GL | IM | 10^2 HAD50 | ASFV-G (Georgia 2007/1) | 10^3 HAD50 | 21 | ASFV-specific antibodies and the presence of ASFV-specific IFN-  -producing cells | 40% | No clinical signs |
|  |  |  |  |  |  |  |  | 28 |  | 100% |  |
|  |  |  |  |  | 10^3 HAD50 |  |  | 21 |  | 100% |  |
|  |  |  |  |  |  |  |  | 28 |  | 100% |  |
|  |  |  |  |  | 10^4 HAD50 | - | - | - | - | - | Severe ASF symptoms |
| O’Donnell et. al (2015b) | Domestic pigs | Attenuated vaccine by genetic manipulation | ASFV-GΔMGF | IM | 10^2 HAD50 | ASFV-G (Georgia 2007/1) | 10^3 HAD50 | 28 | High levels  of circulating anti-ASFV antibodies | 100% | No clinical signs |
|  |  |  |  |  | 10^4 HAD50 |  |  |  |  | 100% |  |
| O’Donnell et. al (2016) | Domestic pigs | Attenuated vaccine by genetic manipulation | ASFV-G-Δ9GL/ΔUK | IM | 10^2 HAD50 | ASFV-G (Georgia 2007/1) | 10^3 HAD50 | 28 | 44,44% ASFV-specific antibody response detected | 44,44% | No clinical signs |
|  |  |  |  |  | 10^4 HAD50 |  |  |  | 100% ASFV-specific antibody response detected | 100% |  |
|  |  |  |  |  | 10^6 HAD50 |  |  |  | 100% ASFV-specific antibody response detected | 93,33% |  |
| Burmakina et. al (2016) | Domestic pigs | Recombinant vaccine and live attenuated vaccine by successive passages in cells | Congo *France*  *CD2v/Lectin*, Congo KK-262, | IM | 10^6 HAU boost 21 dpv | Congo k49 | 10^3 HAU |  | 100 % Ab ASFV ELISA | 20% Congo *France*  *CD2v/Lectin*, 80% Congo KK-262 | - |
|  |  |  |  |  | 2x10^6 HAU boost 21 dpv |  |  |  |  | 0% Congo *France*  *CD2v/Lectin*, 100% Congo KK-262 | - |
|  |  |  | France *Congo CD2v/Lectin*, France FK-32/135 |  | 3x10^6 HAU boost 21,42,63,77 dpv |  |  |  |  | 0% France *Congo CD2v/Lectin*, 20% France FK-32/135 | - |
|  |  |  |  | IM and IV |  |  |  |  |  | 0% France *Congo CD2v/Lectin*, 0% France FK-32/135 | - |
| Carlson et. al (2016) | Domestic pigs | Attenuated vaccine by genetic manipulation | Pret4∆9GL virus | IM | 10^4 HAD50 | Pretoriuskop/96/4 (Pret4) | 10^4 HAD50 | 7 | 0% Ab ASFV ELISA | 40% |  |
|  |  |  |  |  |  |  |  | 10 | 40% Ab ASFV ELISA | 60% |  |
|  |  |  |  |  |  |  |  | 21 | 100% Ab ASFV ELISA | 80% |  |
|  |  |  |  |  |  |  |  | 28 | 80% Ab ASFV ELISA | 80% |  |
| Monteagudo et. al (2017) | Domestic pigs | Attenuated vaccine by genetic manipulation | BA71ΔCD2 | IM | 10^3 PFU | BA71 | 10^3 PFU | 24 | 100% Ab ASFV ELISA and ASFV-specific T cells | 33% | No clinical signs |
|  |  |  |  |  | 3,3x10^4 PFU | BA71 or E75 or Georgia 2007/1 |  |  |  | 100% |  |
|  |  |  |  |  | 10^6 PFU | BA71 or E75 or Georgia 2007/1 |  |  |  | 100% |  |
| Reis et. al (2017) | Domestic pigs | Attenuated vaccine by genetic manipulation | BeninΔDP148R | IM | 10^3 HAD50 and boost 14 dpv | Benin 97/1 | 10^4 HAD50 | 28 | 100% ASFV-specific cellular and humoral immune responses detected | 100% | Transient fever of 1-2 days |
|  |  |  |  |  | 10^3 HAD50 and boost 21dpv |  |  | 42 |  | 100% |  |
|  |  |  |  | Intranasal | 10^3 HAD50 and boost 14 dpv |  |  | 28 |  | 83,33% | Fever and reduced levels of food consumption |
| Sanchez-Cordon et. al (2017) | Domestic pigs | Live attenuated isolated | OURT88/3 | IM | 10^3 TCDI50 | OURT88/1 | 10^4 TCDI50 | 21 |  | 50% |  |
|  |  |  |  |  | 10^4 TCDI50 |  |  |  |  | 66% |  |
|  |  |  |  |  | 10^5 TCDI50 |  |  |  |  | - | Severe ASF symptoms |
|  |  |  |  | Intranasal | 10^3 TCDI50 |  |  |  |  | 100% |  |
|  |  |  |  |  | 10^4 TCDI50 |  |  |  |  | 100% |  |
|  |  |  |  |  | 10^5 TCDI50 |  |  |  |  | 66,66% |  |
| Gallardo et. al (2018) | Domestic pigs | Recombinant vaccine | NH/P68DA238L-COS7, NH/P68DA224L-COS7, NH/P68DEP153R-COS7, NH/P68-PAM | IM | 10^6 TCDI50 | First: Lisboa 60. And then Arm07 | 10 HAD50 | Lisboa 60: 29 dpv. Arm07: 63 dpv |  | Lisboa 60: 100%. Arm07: 100% (NH/P68-PAM), 50% (NH/P68DA224L-COS7), 0% (NH/P68DA238L-COS7, NH/P68DEP153R-COS7) | Clinical signs associated with chronic forms of ASF. E.g.: Swollen joints and necrotic skin areas |
|  |  |  | NH/P68 - PAM |  | 10^6 TCDI50 | Arm07 |  | 30 |  | 100% |  |
|  |  |  | NH/P68DA238L -COS7, NH/P68DA276R-PAM, NH/P68-COS7/PAM |  | 10^2 TCDI50 | Arm07 |  | 29 | 100% Ab ASFV | 100% (NH/P68-PAM), 40% (NH/P68DA238L -COS7), 33% (NH/P68-COS7), 0% (NH/P68DA276R-PAM) |  |
| Sánchez-Cordón et. al (2018) | Domestic pigs | Attenuated vaccine by genetic manipulation | BeninΔMGF | IM | 10^2 TCDI50 and boost 21dpv | Benin 97/1 | 10^4 TCID50 | 39 | 100% Ab ASFV ELISA and ASFV-specific T cells | 50% | Transient increase in temperature was observed  in some immunised pigs for 1 or 2 days |
|  |  |  |  |  | 10^3 TCDI50 and boost 21dpv |  |  |  |  | 66,67% |  |
|  |  |  |  |  | 10^4 TCDI50 and boost 21 dpv |  |  |  |  | 83% |  |
|  |  |  |  | Intranasal | 10^3 TCDI50 and boost 21dpv |  |  |  |  | 66,67% |  |
